# Trismus following different treatment modalities for head and neck cancer: a systematic review of subjective measures

**DOI:** 10.1007/s00405-017-4519-6

**Published:** 2017-03-25

**Authors:** Sook Y. Loh, Robert W. J. Mcleod, Hassan A. Elhassan

**Affiliations:** 1 Cardiff University School of Medicine, Cardiff, UK; 2 Department of Otolaryngology, University Hospital of Wales, Cardiff, UK

**Keywords:** Trismus, Oropharyngeal carcinoma, Surgery, Radiotherapy, Chemotherapy, Quality of life

## Abstract

The aim of this review was to compare systematically the subjective measure of trismus between different interventions to treat head and neck cancer, particularly those of the oropharynx. Using The Preferred Reporting Items for Systematic Review and Meta-Analyses (PRISMA) Guidelines, Six databases were searched for the text using various terms which include “oropharyngeal/head and neck cancer”, “trismus/mouth opening” and the various treatment modalities. Included in the review were clinical studies (> or =10 patients). Three observers independently assessed the papers identified. Among the six studies reviewed, five showed a significantly worst outcome with regard to the quality-of-life questionnaire scores for a radiotherapy or surgery and radiotherapy (RT) ± chemotherapy or chemoradiotherapy when compared to surgery alone. Only one study showed no significant difference between surgery alone and other treatment modalities. Subjective quality-of-life measures are a concurrent part of modern surgical practice. Although subjective measures were utilised to measure post operative trismus successfully, there was no consensus as to which treatment modality had overall better outcomes, with conflicting studies in keeping with the current debate in this field. Larger and higher quality studies are needed to compare all three treatment modalities.

## Introduction

Cancers of the upper aerodigestive tract, collectively known as head and neck cancers, arise from a myriad of sites, including the oral cavity, pharynx, and larynx, as well as the nasal cavity and sinuses [1–3]. Oropharyngeal cancer (OPC) is a rare cancer overall but common in the head and neck region [4]. Head and neck cancer is the sixth most common type of cancer, making up 5.3% of all cases and account for an estimated 348,300 new cancer cases and 179,600 cancer deaths worldwide per year [5]. The median age for diagnosis is around mid-60s, with male predominance, especially for laryngeal cancer [6]. Squamous cell carcinomas (SCC) of varying degrees of differentiation make up the majority of head and neck cancers [2, 3]. About two-thirds of these SCC patients present at an advanced stage, usually with nodal involvement [3].

There is wide discrepancy in treating head and neck cancer. In recent decades, there has been a tendency towards organ preserving treatment [2] and as most head and neck cancers respond well to radiotherapy (at least in the first instance), surgery can be avoided in a high proportion of cases [2]. Chemotherapy has accentuated this organ preserving approach [2]. National Comprehensive Cancer Network (NCCN) guidelines consider concurrent chemoradiation therapy (CCRT) as first-line treatment for oropharyngeal cancers; however, the evidence supporting this is equivocal [7].

All treatment modalities have their pros and cons. Surgery may result in loss of function associated with excision of anatomical structures, whereas radiotherapy may be accompanied by debilitating dysphagia and long-term loss of oral lubrication [2]. Radiotherapy induces fibrosis in the muscles of mastication, leading to trismus, as well as necrosis of bone and soft tissue which restricts mouth opening [8, 9]. Chemotherapy too is associated with significant morbidity and can cause renal dysfunction, ototoxicity, and myelosuppression [2]. Decisions on treatment modality depend on the tumour site and stage, expected functional outcomes and the patients comorbidities and ability to tolerate treatment [3]. There is currently no consensus as to which treatment modality results in better outcomes.

Trismus, which is restricted mouth opening, is common in head and neck cancer patients and interferes with activities, such as eating, swallowing, and speaking [10, 11]. It also interferes with oral hygiene and can be particularly discomforting to patients [12]. Trismus may be caused by tumour infiltration into the masticatory muscles, specifically the pterygoids, or temporomandibular joint (TMJ) or induced by cancer treatment, including surgery and/or radiotherapy(RT) [13, 14]. Post-treatment trismus is unpredictable in both its frequency and severity [12] and usually develops 3–6 month post-radiotherapy and often becomes a lifelong problem [13].

Prevention of trismus is more desirable than treating it [14]. Patients at risk of trismus should have home exercises to maintain maximum mouth opening and jaw mobility as soon as they start radiotherapy [15, 16]. Patients who develop trismus require an intensive exercise programme, and if necessary, combined with physiotherapy to improve mouth opening [15]. Prosthetic appliances (dynamic bite openers) containing springs and bands are able to re-stretch the muscles to help patients suffering from trismus [15, 16]. Patient concordance with trismus exercises is paramount if preventative and treatment regimes are to be successful [17].

## Review aim

Trismus can be measured both objectively and subjectively. The goal of this review is to compare the different tools for the subjective measures of trismus and assess the most suitable questionnaire to be used for measuring trismus. We include a systematic literature review to compare the effect of different head and neck cancer treatment modalities on trismus using subjective measures encountered.

## Objective measures of trismus

Normal mouth opening varies between individuals, within a range of 40–60 mm [18]. Males generally display greater mouth opening than females [19]. Maximal interincisal opening (MIO) is the maximal distance between the edges of the upper and lower incisors and is widely used to objectively measure trismus [8, 10, 11, 20–23]. For edentulous patients, the distance from one alveolar ridge to the opposing side vertically can be used instead [14]. These measurements can be made using callipers or other devices [11, 20]. The MIO should be measured before treatment is commenced, and the patient or clinician should measure this distance frequently post-treatment to ensure its maintenance [17]. As there is no agreed absolute measurement of trismus in clinical practice, studies have mostly used a cut-off of ≤35 mm [10, 11, 21–26].

## Subjective measure of trismus

Patients may continue to experience trismus in spite of objectively ‘normal’ MIO. The emergence of the patient reported outcome measures (PROMs) and quality-of-life questionnaires allows clinicians to look beyond mortality as the sole outcome measure of successful medical interventions. Subjective measures can also be used to compare different treatments [27]. Such subjective measures of trismus are vital in managing this condition.

Our review identified several questionnaires that address trismus (Table 1). These subjective trismus measures consist of either a mouth opening specific questionnaires, such as the Mandibular Function Impairment Questionnaire (MFIQ) [21], or a subset question in a general cancer quality-of-life questionnaire, such as the Performance Status Scale (PSS) [28].

**Table 1:** Summary of questionnaires

Mouth-opening specific questionnaires	General quality-of-life questionnaires
Mandibular Function Impairment Questionnaire (MFIQ) [21]	European Organization for Research and Treatment of Cancer (EORTC) QLQ-C30 questionnaire [7, 8, 23, 24, 27, 29–32]
The Liverpool Oral Rehabilitation Questionnaire (LORQ v3) [26, 33]	EORTC QLQ-H&N35 questionnaire [7, 8, 23, 24, 27, 29–32]
Gothenburg Trismus Questionnaire (GTQ) [20]	Performance Status Scale (PSS) [28]
	The University of Washington quality-of-life scale (UWQOL v4) [23, 26]
	Functional Assessment of Cancer Therapy—Head and Neck Scale (FACT-H&N) [34]

There is a suggested definition of severe trismus with a PSS score of ≤50, European Organization for Research and Treatment of Cancer (EORTC) QLQ-C30 and EORTC QLQ-H&N35 ≥50 [28].

## Mouth-opening specific questionnaires

The first study that aimed to determine the cutoff for trismus used the Mandibular Function Impairment Questionnaire (MFIQ) [21]. This questionnaire contains 11 items which assessed perceived difficulties in mandibular function during social activities, speaking, taking large bites, chewing hard food, chewing soft food, work and/or daily activities, drinking, laughing, chewing resistant food, yawning, and kissing [21]. There were also six other items which took into account difficulties during eating specific foods, i.e., a hard cookie, meat, a raw carrot, French bread, peanuts/almond, and an apple [21]. Answers were scored (0) no difficulty, (1) a little difficulty, (2) quite a bit of difficulty, (3) much difficulty, and (4) very great difficulty or impossible without help [21]. Scores were summed up, with a range between 0 and 68 and a high score indicating more impairment [21]. The internal consistency of the questionnaire lies between 0.80 and 0.95 [29].

The Liverpool Oral Rehabilitation Questionnaire (LORQ v3) comprises 40 items, whereby 17 relate to oral function, oro-facial appearance, and social interaction [30]. Five questions were included to assess the impact of chewing ability on social life and choice of food: (1) did you experience difficulty with chewing? (2) did you have pain when you chew? (3) did your chewing ability affect your social life? (4) did your chewing ability influence your choice of foods? and (5) did you experience difficulty with mouth opening? [26] Options for an answer included ‘Always’, ‘Often’, ‘Sometimes’, or ‘Never’ [26].

The Gothenburg Trismus Questionnaire (GTQ) is a trismus specific questionnaire with good psychometric properties (validity and reliability), but has only been used in one study [20]. This questionnaire contains 21 items with 13 items divided into the three domains: jaw-related problems (six items); eating limitations (four items); and muscular tension (three items) [20]. The domains and single items range from 0 to 100; 100 indicating maximal amount of symptoms and 0 being symptom free [20]. The GTQ has a 1 week recall period for the three domains [20]. Patients with trismus reported more health-related quality-of-life impairments in the domains of mouth opening (*p* < 0.001), jaw-related problems (*p* < 0.05), eating limitations (*p* < 0.05), and muscular tension (*p* < 0.001) [20]. These results were in line with the incidence of trismus, and are compatible with results from other studies [8, 20, 31]. The GTQ has been suggested as a screening tool and for evaluating endpoints in intervention for jaw physiotherapy and rehabilitation studies [20].

## General quality-of-life questionnaires

The PSS consists of three subscales, such as eating in public, normalcy of diet, and intelligibility of patients speech [28, 32]. Each is rated from 0 to 100, whereby higher scores suggest better performance [32].

The UWQOL v4 is validated in the field of head and neck cancers but is not as widely used as the EORTC questionnaires [26]. It assesses 12 domains: pain, appearance, activity, recreation, swallowing, chewing, speech, shoulder function, taste, saliva, mood, and anxiety, whereby the chewing, saliva, mood, and anxiety domains which are measured on a Likert scale from 0 (worst) to 100 (best) were included in the study questionnaire to identify the effects of limited mouth opening on these domains [26]. There was no mention of a scoring system to define trismus using the UWQOL [26].

The FACT uses means of self-reporting and comprises 28 general + 11 head and neck specific items, rated from 0 to 4 on a Likert-type scale [32]. The FACT domains describe function in six areas: physical well-being, social and family well-being, relationship with doctor, emotional well-being, functional well-being, and head- and neck-related symptoms (HNS) [32, 33]. The FACT-H&N module includes additional concerns, such as oral comfort, breathing, voice, eating, appearance, tobacco, alcohol, and communication [33].

The EORTC developed an established system assessing the health related quality of life (QOL) of head and neck cancer patients using two questionnaires, the general EORTC QLQ-C30 questionnaire, and the head- and neck-specific EORTC QLQ-H&N35 module which subjectively measured mouth opening among the six scales [34]. The EORTC QLQ-C-30 is a self-assessment of health-related QOL for patients with cancer, including difference function scores, a score for global QOL, and symptoms scores relevant for cancer patients [28, 35]. The five functional scales are physical, role, emotional, cognitive, and social functioning, while the six symptom scores include dyspnoea, insomnia, appetite loss, constipation, diarrhoea, and financial difficulties [28]. The supplementary EORTC QLQ-H&N35 consists of 35 additional questions to assess head and neck cancer-related symptoms (7 multi-item scales and 11 single items, validated in a sample of 500 patients from Norway, Sweden, and The Netherlands) [28, 35, 36]. Scales and single questions are scored on categorical scales and linearly converted to a scale of 0-100 [35]. A score of 100 on the functioning scales and the Global QOL scale represents maximal functioning, while a score of 100 on the symptom scales and single items indicate worst possible symptoms [20]. Changes in the score of >10 points over time could be clinically significant [20]. The multi-item scales consist of pain, swallowing, senses, speech, social eating, social contact, and sexuality, while the single items are teeth, mouth opening, dry mouth, sticky saliva, coughing, feeling ill, pain killers, nutritional supplements, feeding tube, weight loss, and weight gain [28]. High function scores and a low symptom scores correlate to good functioning and few symptoms [35]. The EORTC has been used worldwide, including a validated Chinese version of the EORTC core questionnaire and head and neck module [37]. These questionnaires have been continuously re-evaluated and proven to be sensitive and comprehensive [38, 39].

## Correlation between subjective and objective trismus measures

A couple of studies used both objective and subjective measurements for trismus [20, 23, 26]. Correlation between objective measurements of mouth opening and subjective measures using the UWQOL questionnaire has been attempted. This identified a significant association of mouth opening on the UWQOL chewing domain (*r*_s_ = 0.45, *p* < 0.0001) and in the UWQOL overall quality of life (*r*_s_ = 0.25, *p* = 0.01) [26]. However, there were exceptions whereby 16 patients with objective trismus denied problems with mouth opening while ten patients who were not shown clinically to have trismus complained subjectively of problems with mouth opening [26]. Despite this, correlation between subjective and objective measurements was overall strong.

Other studies that used subjective or objective measures have had comparable finding. Studies using objective measures found that patients who received adjuvant radiotherapy and multi-modality treatments had worst outcomes than those treated with surgery alone, in line with studies measuring trismus subjectively [8, 22, 25, 26, 40, 41]. The difference in mean MFIQ scores for patients with trismus and without trismus for a cut-off point of ≤35 mm was significant [21]. An MFIQ score of 8.3 was the minimum score of for trismus, equivalent to a mouth opening of 35 mm [21].

## Materials and methods

### Search strategy

The preferred reporting items for systematic review and meta-analyses (PRISMA) guidelines were used to perform a systematic review of available literature. Studies evaluating the effects of different types of head and neck cancers, especially the oropharyngeal region on mouth opening, were searched for using PubMed, Cochrane Library, Science Direct, Scopus, EMBASE (1947–Present) and Ovid MEDLINE (1946 to May Week 2 2015). Four search domains were used and combined using “AND”, while terms within each domain combined by “AND/OR”. The keywords “head and neck cancer”, “SCC oropharynx”, “oropharyngeal cancer”, “oropharyngeal carcinoma”, “SCC base of tongue”, “tonsil SCC”, “oropharyngeal neoplasm”, “oropharyngeal tumor”, “oropharyngeal tumour”, and “cancer of the oropharynx” were used in the first domain. The second domain encompassed “mouth opening”, “jaw opening”, and “trismus”. Finally, the terms “surgery”, “radiotherapy”, “radiation therapy”, “chemotherapy”, “chemoradiotherapy”, “chemoradiation”, “chemotherapy and radiotherapy”, “chemotherapy and radiation therapy”, and “transoral robotic surgery” were used in the third domain. The final domain consisted of “quality of life”.

**Fig. 1:**
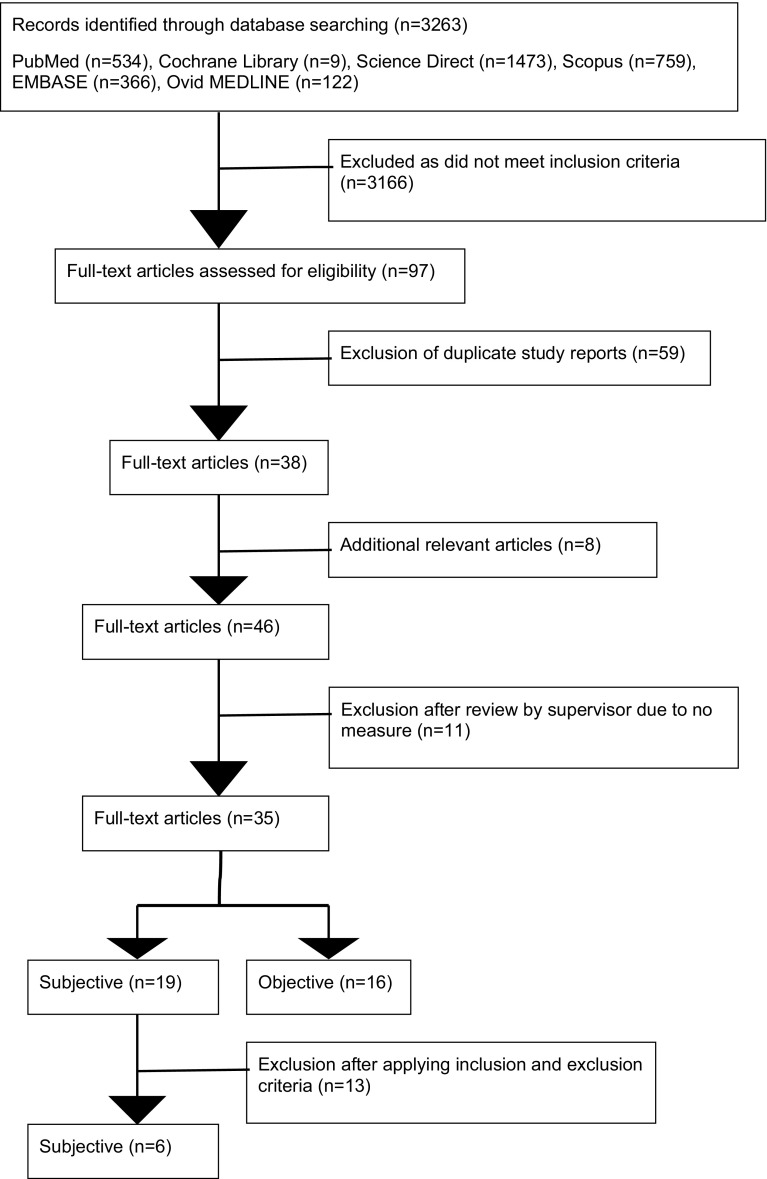
Trismus search strategy to obtain literature using PRISMA guidelines

The primary search identified 3243 records. 3146 of these were excluded following screening. 59 of the remaining studies were duplicates, thus leaving 38 studies for full text evaluation. Eight studies were added through cross-referencing. Following application of our inclusion and exclusion criteria to these full texts, a further 11 studies were excluded. A total of 35 studies were included, 19 involving subjective measures of trismus, while the other 16 only measured trismus objectively. Six subjective studies, which compared treatment modalities, were chosen for the final review (Fig. 1).

### Inclusion and exclusion criteria

Original journal articles that studied the effects of oropharyngeal carcinoma treatment on mouth opening as assessed using qualitative measurements were included. Eligible, studies needed to be carried out on humans, involve ten or more patients, included post-treatment measurements, and included treatment with surgery, radiotherapy (RT), chemotherapy, or a combination of any of the three modalities. Articles were excluded if there was no patient involvement, unrelated to head and neck oncology, written in languages other than English, and focused on dental hygiene during oncology treatment or primarily discussed exercise regimes to treat trismus. Letters to the editor, case reports/series (<10 participants) and poster abstracts were also excluded.

## Results

The questionnaires used in the studies included in the systematic review were the EORTC QLQ-C30 and EORTC QLQ-H&N35 [7, 27, 42–45]. None of these studies used objective measures to measure trismus. Table 2 shows a summary of the studies that were included in this review.

**Table 2:** Summary of the studies included

Study	Study type	No. of patients	Inclusion criteria	Exclusion criteria	Questionnaire used	QoL significance	Follow up
Ryzek et al. (2014)	Retrospective cohort study	111	(1) tumor was located within the oropharynx (2) primary tumor was of early stage (pT1 or pT2, N0-2) (3) no distant metastasis were detected (M0) (4) tumor was successfully treated with a minimum tumor free interval of 18 months after treatment considered were surgery alone (OP) or a combination of surgery and radio- and/or chemotherapy (OPRT/OPRCT)	All patients with a recurrent disease	Patients’ QOL evaluated in detail using the German-language versions of two standardised questionnaires from the European Organization for Research and Treatment of Cancer (EORTC), specifically the Core Module (EORTC-QLQ-C30) and the Head and Neck Cancer Module (EORTC-QLQ-H&N35)	Not stated	Not stated
Van Cann et al. (2005)	Retrospective cohort study	105	Undergone marginal or segmental mandibular resection for oropharyngeal squamous cell carcinoma (oscc), adjacent or fixed to the mandible Operated between 1995 and 2000	Patients who had had previous malignancies	EORTC-QLQ-C30 & EORTC-QLQ-H&N35 questionnaires	For global health status and functional scale, the best is 100, for symptom scales, the best is 1	Ranged from two to seven years
Tschudi et al. (2003)	Retrospective (chart review) study	217 (treated with curative intent between January 1990 and December 1998)	Consecutive patients between January 1990 and December 1998 with previously untreated oropharyngeal carcinoma treated with curative intent by surgery or radiation therapy alone or of surgery followed by postoperative adjuvant irradiation In January 2001, a total of 111 disease-free survivors were identified and included in this study	Recurrent disease or secondary primary tumours	EORTC-QLQ-C30 & EORTC-QLQ-H&N35 questionnaires	As before	At least 2 years
Boscolo-Rizzo et al. (2009)	Retrospective, cross-sectional study	57	(1) patients with previously untreated T3–T4 OC (2) complete remission after surgery plus PORT or CRT (3) treatment completed at least 24 months prior to inclusion in the study	Not stated	EORTC-QLQ-C30 & EORTC-QLQ-H&N35 questionnaires	As before	Median follow-up for surviving patients was 56 months (range, 11–124)
Infante-Cossio et al. (2009)	Long-term prospective longitudinal (QoL) study (carried out between January 2000 and December 2001)	128	Patients who had recently been diagnosed with squamous cell oral and oropharyngeal carcinoma, and who were admitted for treatment Oral cavity tumours included those located on the mobile tongue, gums, floor of the mouth, buccal mucosa, hard palate and buccal area of the soft palate Oropharyngeal tumours were located behind the anterior pillar of the pharynx, retromolar trigone, tonsils, tonsillar region of the soft palate and base of tongue	Cases that had not been confirmed through biopsy Those presenting with poor general condition, serious concomitant disease, mental or psychomotor disorders preventing the interview with the physician or patients with a previous history of cancer, local or distant recurrences	EORTC-QLQ-C30 & EORTC-QLQ-H&N35 questionnaires	Scores of all these scaled were transformed on to a 0-100 scale	Pre-treatment, 1 and 3 years post-treatment
Kim et al. (2010)	Cross-sectional (retrospective) study	133	From 1995 to 2007, patients who were diagnosed with oropharyngeal cancer in the Department of Otorhinolarngology-Head and Neck Surgery at Samsung Medical Center	Double or multiple primary cancers Referral after any curative-intent treatment A proven pathology other than squamous cell carcinoma or an incomplete medical record	QOL questionnaire were completed by telephone interview The Korean version of the European Organization for Research and Treatment of Cancer Quality-of-Life Questionnaire (EORTC QLQ) validated by Yun et al EORTC-QLQ-C30 & EORTC-QLQ-H&N35 questionnaires	As before	Ranged from 1 to 155 months (average 43.7 months)

Ryzek et al. showed significantly better results among early stage oropharyngeal cancer (OPC) for the surgery-only treatment group when compared to either surgery combined with RT or surgery combined with any type of adjuvant therapy for mouth opening (*p* ≤ 0.05) in the EORTC-QLQ-HN35 [27].

A further study evaluating OPC patients using the EORTC QLQ-H&N35 questionnaire [43] found that radiation therapy significantly augmented the patient’s complaint about restricted mouth opening (surgery versus radiation, *p* = 0.008; surgery versus surgery plus irradiation, *p* = 0.0008) [43]. When patients undergoing surgery ± RT were compared with the non-surgically treated group (radiation therapy alone), patients in the no surgery group suffered significantly more from restricted mouth opening (*p* = 0.03) [43]. Comparison between patients receiving any kind of radiation therapy ± surgery and those treated with surgery alone, radiation therapy leads to significantly more problems with mouth opening (*p* = 0.001) [43].

Kim et al. investigated OPC cancer patients and found that there was no significant difference in mouth opening between the surgery-based group and the RT-based group (*p* = 0.9024) [7].

Boscolo-Rizzo et al. analysed OPC patients and found that the chemoradiotherapy (CRT) group reported significantly greater problems with mouth opening (*p* = 0.036, mean difference 18.1) when compared with those who had surgery + postoperative RT [44].

Postoperative radiotherapy had a significant correlation to impact mouth opening on the EORTC QLQ-H&N35 symptom scale among patients who had undergone marginal or segmental mandibular resection for oral and oropharyngeal squamous cell carcinoma (OSCC) (*p* = 0.003, *p* ≤ 0.003) [42].

Patients who had recently been diagnosed with squamous cell oral and oropharyngeal carcinoma, who underwent surgery combined with adjuvant RT and chemotherapy presented worse evaluations of QoL, more affectation of less oral opening compared to those who had surgery alone [45].

## Discussion

Subjective quality-of-life measures are a concurrent part of modern surgical practice. Ideally, these questionnaires should be validated [27]. Other QOL questionnaires, such as the mandibular function impairment questionnaire (MFIQ) [21] and Performance Status Scale (PSS) [28], have not been validated, while Van Der Molen et al. used a study-specific questionnaire for QOL evaluation [46].

The European Organization for Research and Treatment of Cancer have shown a great interest in module development. A module may assess (1) the disease symptoms related to a specific tumour site (e.g., abdominal pain in colorectal cancer), (2) the side-effects relating to a specific treatment (e.g., radiotherapy-induced skin problems), or (3) additional QoL domains affected by the disease of treatment (e.g., sexuality, body image and future perspective) [47]. The developmental process of these questionnaires is subjected to internal peer review to ensure uniformly high-quality modules. These sets of questionnaires have undergone three phases of module development, psychometric performance, and cross-cultural validity [47]. The multinational, cross-cultural, and multidisciplinary composition of the EORTC study group have enabled crucial scientific and cultural input to the development of the modules [47]. These are among the reasons that the EORTC QLQ sets of questionnaires have been extensively used in studies to measure trismus in head and neck cancer. The LORQ questionnaire has showed promising abilities to assess oral rehabilitation in patients with oral and oropharyngeal cancer, showing good construct validity and reliability in a pilot study [48]. It was used alongside the UWQOL and the EORTC QLQ questionnaires to determine its validity and reliability [48]. However, the study size was small and future studies might have to look into analysing this questionnaire with a larger cohort [48].

Although the EORTC QLQ-C30 and the QLQ-H&N35 have been used more frequently, it is undeniable that the GTQ questionnaire is a potential successor as it was found to be useful in identifying patients’ change in functional mouth opening over time [20]. As for now, the EORTC QLQ sets of questionnaires are the set standard for a thorough, valid, and reliable method to determining quality of life in head and neck cancer patients.

### Surgery

Nonsurgical treatments, deemed less invasive, are assumed to lead to better QoL outcomes [44, 49]. However, Ryzek et al. using the EORTC questionnaire for mouth opening reported higher scores among surgery-only patients when compared to surgery plus RT or any adjuvant therapy [27].

Patients undergoing surgery ± RT had less restricted mouth opening, using subjective measures, than the non-surgically treated patients. In this group, primary surgical resection achieved the highest QoL score in the head and neck specific EORTC QLQ-H&N35 module of the three treatment modalities [43]. However, careful interpretation is required as these patients presented with a lower initial tumour stage than those treated with primary radiotherapy or surgery plus adjuvant radiotherapy [43]. We have included Trans Oral Robotic Surgery (TORS) in our search, but it did not yield any trismus related PROMs or studies.

### Radiotherapy

The EORTC QLQ-C30 and H&N35 used to subjectively measure trismus revealed that non-irradiated patients had significantly less trouble with mouth opening compared to those treated with either primary or postoperative radiation therapy [43]. However, mouth opening was not significantly different between surgery-based and RT-based treatment in a study by Kim et al. who also used the EORTC QLQ-C30 and H&N35 [7, 43].

### Chemotherapy

Positive predictive factors of trismus include treatment with concurrent chemoradiotherapy [50]. The CRT treatment group reported significantly greater problems with trismus than surgical patients using EORTC QLQ-C30 & QLQ-HV35 [44], where patients with T3-4 oropharyngeal cancer after surgery plus postoperative RT (26 patients) were compared to versus CCRT (31 patients). Conflicting results, using the subjective measures do arise, as seen by Payakachat et al. when using the EORTC QLQ-H&N35, the median score differences on the open mouth item were significantly higher in the Surgery + RT group when compared with the CRT group [51].

### Multimodality treatment

Few studies compare QOL outcomes in different treatment modalities of oropharyngeal cancers [52, 53]. The studies that do look at QOL outcomes do not specifically look into the aspect of mouth opening [52, 53]. Only Infante-Cossio et al. showed that patients who underwent surgical treatment combined with adjuvant radiotherapy and chemotherapy generally showed a worse score for mouth opening, needing a longer recovery time compared to surgery alone and surgery plus RT [45].

### Tumour staging

One study showed that patients in stages III and IV had a worse evaluation of their state of health and QoL, showing a higher incidence of pain, tiredness, less appetite, more swallowing problems, speech problems and problems with social contacts and eating in public, limited oral opening cough, weight loss, and more analgesia consumption [45]. There is a significant association between tumour staging and QoL problems [41, 54–56]. Patients with early stage cancer showed better overall QoL, both at the beginning and after 1 year, than those at more advanced stages [45].

## Trismus study design

**Fig. 2:**
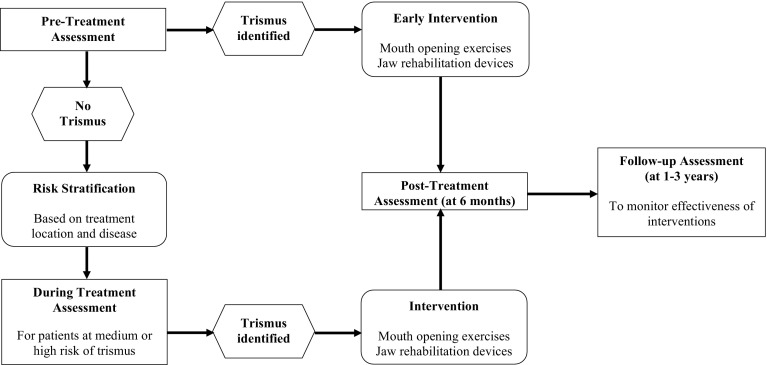
Algorithm for measuring trismus in future studies. Assessment to include the following: *Objective* use callipers or similar instrument to measure the inter-incisor distance. Document in patient notes and/or database. *Subjective* use questionnaire (EORTC QLQ-C-30 and EORTC QLQ-H&N35) to score patients’ reported perception of trismus. Questionnaire added to patients’ notes and/or database

As per guidelines, mouth opening should be measured throughout treatment. For the purposes of future research, we have devised an algorithm for future studies to demonstrate when to measure for trismus at the various stages of the patients’ treatment and follow-up (Fig. 2). This algorithm includes minimum points for trismus assessment and data capture, i.e., at the time of diagnosis, post treatment, and after rehabilitation. We suggest using the EORTC set of questionnaires, which are the EORTC QLQ-C30 and the EORTC QLQ-H&N35 as they were the most established and were used in the studies included in our systematic review. We suggest a further trismus assessment 1 year post-treatment as studies report no further changes in health-related quality of life after the first postoperative year [57]. We suggest that patients should be followed up for at least 3 years [45].

## Limitations

Our systematic review of subjective outcome measures contained studies with small cohorts. Furthermore, the existence of numerous confounding factors when comparing different treatment modalities, which cannot be eliminated are an ongoing limitation while utilising subjective trismus measures [27]. Other limitations include lack of information on the relationship between objective and subjective measures in most studies, hence not being able to ascertain the reliability of other questionnaires in practice. There are also variations between questionnaires in how they determine QoL with regard to the type of questions asked and also indirect parameters that suggest or can cause difficulty in mouth opening, such as chewing, speech, and pain. Some studies also focused on certain types of cancer, such as oropharyngeal cancer, while others took into account generally all cancers of the head and neck.

## Conclusion

Subjective measures were utilised to measure post-operative trismus successfully. While cure rates are given the greatest priority and treatment if guided by the UICC grade, subjective measures, such as patient, reported outcome measures (PROMs) and QoL questionnaires are important and useful tools for assessing patient well-being. This is particularly important in head and neck cancer, where the treatment modalities result in equal effectiveness.

These subjective measures, QoLs and PROMs, are also of use in monitoring side effects of treatment, e.g., trismus, so that early intervention can be implemented to treat or prevent progression. This manuscript demonstrates that future head and neck cancer treatment studies must incorporate functional measurements together with QOL measurements, because functional improvements alone do not correlate to a perceived improvement in trismus outcomes according to patients [7]. We envisage future studies prospectively performing objective and subjective measures of mouth opening prior, during and after treatment. Subjective measures may be early indicators of developing trismus, which would have implications for early intervention.
